# Marker Assisted Forward Breeding to Combine Multiple Biotic-Abiotic Stress Resistance/Tolerance in Rice

**DOI:** 10.1186/s12284-020-00391-7

**Published:** 2020-05-29

**Authors:** Shilpi Dixit, Uma Maheshwar Singh, Arun Kumar Singh, Shamshad Alam, Challa Venkateshwarlu, Vishnu Varthini Nachimuthu, Shailesh Yadav, Ragavendran Abbai, Ramchander Selvaraj, M. Nagamallika Devi, Perumalla Janaki Ramayya, Jyothi Badri, T. Ram, Jhansi Lakshmi, G. Lakshmidevi, Jai Vidhya LRK, Ayyagari Phani Padmakumari, G. S. Laha, M. S. Prasad, Malathi Seetalam, Vikas Kumar Singh, Arvind Kumar

**Affiliations:** 1 International Rice Research Institute (IRRI), South-Asia Hub, ICRISAT, Hyderabad, India; 2 International Rice Research Institute, South Asia Regional Centre (ISARC), Varanasi, 221006 India; 3 Leibniz Institute of Plant Genetics and Crop Plant Research (IPK), Gatersleben, Germany; 4 ICAR-Indian Institute of Rice Research (IIRR), Rajendranagar, Hyderabad, India; 5 Professor Jayashankar Telangana State Agricultural University (PJTSAU), RARS, Warangal, India

**Keywords:** Marker assisted breeding, Forward breeding, Pyramiding, Genes, Drought, Disease, Pest, Resistance, Abiotic, Biotic

## Abstract

****Background**:**

Unfavorable climatic changes have led to an increased threat of several biotic and abiotic stresses over the past few years. Looking at the massive damage caused by these stresses, we undertook a study to develop high yielding climate-resilient rice, using genes conferring resistance against blast (*Pi9*), bacterial leaf blight (BLB) (*Xa4, xa5, xa13, Xa21*), brown planthopper (BPH) (*Bph3, Bph17*), gall midge (GM) (*Gm4, Gm8*) and QTLs for drought tolerance (*qDTY*_*1.1*_ and *qDTY*_*3.1*_) through marker-assisted forward breeding (MAFB) approach.

****Result**:**

Seven introgression lines (ILs) possessing a combination of seven to ten genes/QTLs for different biotic and abiotic stresses have been developed using marker-assisted selection (MAS) breeding method in the background of Swarna with drought QTLs. These ILs were superior to the respective recurrent parent in agronomic performance and also possess preferred grain quality with intermediate to high amylose content (AC) (23–26%). Out of these, three ILs viz., IL1 (*Pi9*+ *Xa4*+ *xa5*+ *Xa21*+ *Bph17*+ *Gm8*+ *qDTY*_*1.1*_+ *qDTY*_*3.1*_), IL6 (*Pi9*+ *Xa4*+ *xa5*+ *Xa21*+ *Bph3*+ *Bph17*+ *Gm4*+ *Gm8+ qDTY*_*1.1*_+ *qDTY*_*3.1*_) and IL7 (*Pi9+ Xa4*+ *xa5*+ *Bph3*+ *Gm4*+ *qDTY*_*1.1*_+ *qDTY*_*3.1*_) had shown resistance\tolerance for multiple biotic and abiotic stresses both in the field and glasshouse conditions. Overall, the ILs were high yielding under various stresses and importantly they also performed well in non-stress conditions without any yield penalty.

****Conclusion**:**

The current study clearly illustrated the success of MAS in combining tolerance to multiple biotic and abiotic stresses while maintaining higher yield potential and preferred grain quality. Developed ILs with seven to ten genes in the current study showed superiority to recurrent parent Swarna+drought for multiple-biotic stresses (blast, BLB, BPH and GM) together with yield advantages of 1.0 t ha^− 1^ under drought condition, without adverse effect on grain quality traits under non-stress.

## **Background**

Climatic changes arising because of global warming, deforestation during the past few years has drastically affected the world scenario which has ultimately led to increased threat to our ecosystem (Bellard et al. 2012). This has not only affected humans, animals, birds, reptiles, plants/trees but also pests and pathogens. Crop losses due to unfavorable climatic changes are majorly due to increased incidences of several abiotic and biotic stresses over the past few years. An integrated approach to introgress important genes/QTLs conferring resistance/tolerance against major biotic and abiotic stresses together with selection for high yield under non-stress and reduced disease infestation/yield loss under biotic and abiotic stresses respectively will assist to combat with this situation (Pang et al. 2017; Jena et al. 2017; Wang et al. 2017; Das et al. 2018; Kumar et al. 2018; Sandhu et al. 2019; Muthu et al. 2020).

Rice grain yield is significantly affected by multiple stresses in most parts of the world, especially in Asia and Africa. Abiotic stresses such as drought, salinity, high temperature, cold and heavy metals have a negative impact on growth and development of rice plants. The abiotic stresses can cause yield reduction up to 70% by adversely affecting rice survival, growth and grain filling depending upon the time of occurrence of these abiotic stresses (Akram et al. 2019). Similarly, biotic stresses include pathogens (fungi and bacteria), insects, pests and weeds etc. impart severe yield losses or crop failure during infestation (Hasan et al. 2015). In rice, the major biotic stresses which result in yield penalty are blast, BLB, BPH and GM. Breeding rice varieties with enhanced resistance against multiple stresses with good agronomic yield potential and better rice grain quality is the need of the hour since decades (Khush 2005). In this context, genetic improvement is the most effective solution that enables reducing the adverse effect on the environment through reduced usages of pesticides which directly or indirectly affect our ecology and ecosystem. Recent advances in breeding to transfer genes/QTLs with high precision has provided new opportunities to combat challenges arising from different biotic and abiotic stresses (Kumar et al. 2018).

Blast is one of the most serious and most devastating diseases caused by fungal pathogen *Magnaporthe oryzae,* which is a major threat to global food security (Khush and Jena 2009). The pathogen infects rice crop at every stage of its growth starting from nursery to grain filling stage. Symptoms of the disease can be seen as lesions on plant parts above the ground level resulting in yield reduction up to 50% (Babujee and Gnanamanickham 2000). Till date, more than 100 blast resistance (R) genes and about 500 quantitative trait loci (QTLs) have been identified (Ashkani et al. 2015). Out of them, 28 resistant genes have been cloned and characterized (Das et al. 2012; Wu et al. 2015; Zheng et al. 2016). *Pi9* is a major resistant gene which offers broad spectrum resistance against diverse isolates of *M. oryzae* (Qu et al. 2006) and was identified from the wild species *Oryza minuta* (Sitch et al. 1989; Amante-Bordeos et al. 1992). Introgression of blast gene *Pi9* to develop Pusa 1637-18-7-6-20 demonstrated its superiority over the lines introgressed with *Pi2* gene and recurrent parent Pusa Basmati 1(Khanna et al. 2015).

Bacterial leaf blight (BLB), caused by *Xanthomonas oryzae pv. oryzae* is another very important and destructive disease in rice which results in 20–40% yield reduction at a maximum tillering stage while infection at initial stage causes yield loss of about 50% (Yasmin et al. 2017). To date, 43 genes conferring resistance against BLB have been identified (Busungu et al. 2016; Dilla-Ermita et al. 2017) and only a few of them have been used for the development of resistant lines using MAS. It is reported that single resistant genes for BLB cannot provide durable resistance against the prevalent pathotypes of *Xoo* in India. Using a combination of 3 or 4 genes is broadly effective throughout India (Yugander et al. 2017). Pyramiding of *xa5*+ *xa13*+ *Xa2*1 (Singh et al. 2001; Kottapalli et al. 2010; Pradhan et al. 2015; Dokku et al. 2013a, 2013b), *xa13*+ *Xa21* (Arunakumari et al. 2016, Swathi et al. 2019, Joseph et al. 2004), *Xa4*+ *xa5*+ *Xa21* (Jeung et al. 2006 and Suh et al. 2015) and *Xa4*+ *xa5*+ *xa13*+ *Xa21* (Das and Rao 2015) has been reported to provide durable BLB resistance.

Brown planthopper (BPH) is the most devastating insect of rice. Many resistant genes to BPH have been identified (*Bph1*, *Bph3*, *Bph14, Bph15*, *Bph17*, *Bph18*) which provide resistance against this insect. Successful introgression of *Bph14* and *Bph15* (He et al. 2019; Wang et al. 2019); *Bph3* (Qing et al. 2019) have been reported to provide higher levels of resistance in the recurrent parent. GM is another serious pest of rice in India, which results in huge yield reduction because of formation of silver shoots (Bentur et al. 2003). Several gall midge resistance genes have been identified (*Gm1*, *Gm2*, gm3, *Gm4*, *Gm5*, *Gm6*, *Gm7*, *Gm8, Gm9, Gm10* and *Gm11*) and used in breeding rice against different biotypes of gall midge. This includes introgression of gall midge resistance genes *Gm4* and *Gm8* for improvement of RPHR-1005 through marker-assisted backcross breeding (MABB) (Kumar et al. 2017) and introgression of *Gm1* and *Gm4* genes along with eight more genes/QTLs for different traits in Improved Lalat using MABB (Das and Rao 2015).

Swarna*+*drought (*qDTY*_*1.1*_ and *qDTY*_3.1_) is a high yielding late duration (130 days) drought-tolerant near-isogenic line (NIL) in the background of Swarna developed at IRRI, Philippines using MAS approach. This NIL was found to be susceptible to blast, BLB, BPH, and GM. In this context, we planned to introgress the *Pi9* gene for blast; *Xa4, xa5, xa13* and *Xa21* genes for BLB; *Bph3* and *Bph17* genes for BPH and *Gm4*, *Gm8* genes for GM in NIL Swarna+drought which possess two drought QTLs (*qDTY*_*1.1*_ and *qDTY*_*3.1*_). The MAFB approach was utilized to combine the above mentioned genes/QTLs from five different donor parents in the Swarna+drought background.

## **Results**

### **Parental Polymorphic Survey**

The MAFB approach was utilized to track the allele of interest through foreground selection of single plants (SP) to combine all the targeted genes/QTLs. A set of known functional markers for *xa5, xa13, Xa21, Gm4, Gm8* genes, linked marker for *Xa4* gene and peak /flanking SSR marker for *Bph3, Bph17* genes and *qDTY*_*1.1*_*, qDTY*_*3.1*_ QTLs were used for selection of the respective genes/QTLs (Table S1). In the case of linked markers, only those markers were selected for foreground selection which showed > 10 bp difference between recurrent and donor parents (Table S2-S3). The polymorphic markers specific for the targeted genes/QTLs were utilized to select the desired recombinant in each generation.

### **Forward Breeding Approach to Pyramid Multiple Stress Responsive Genes/QTLs**

**Fig. 1:**
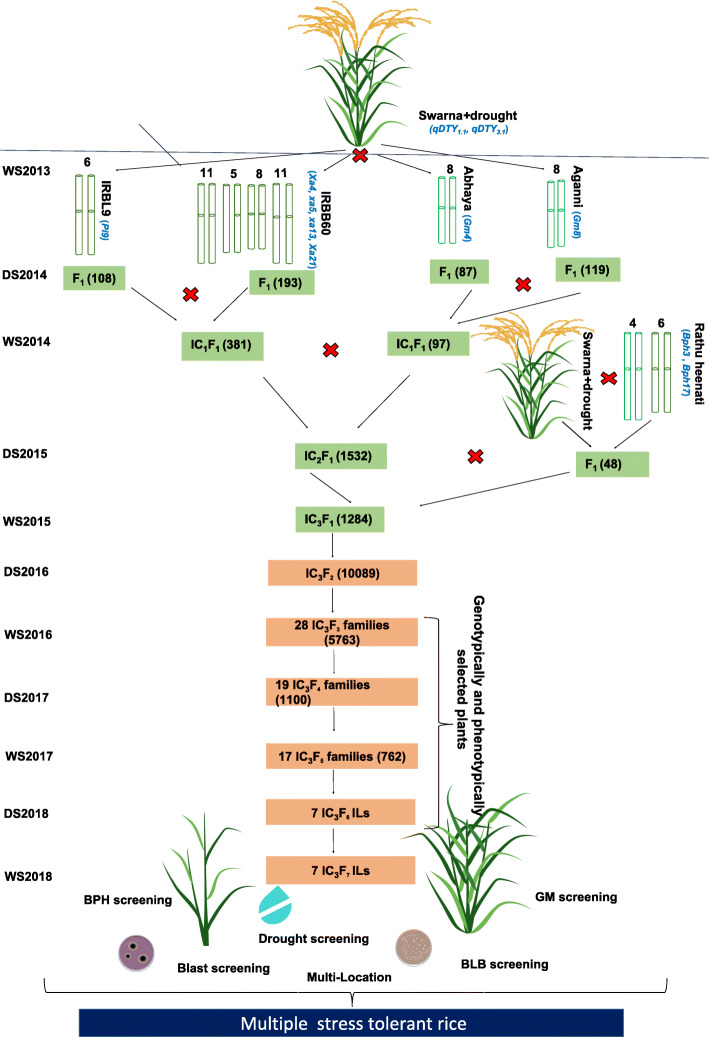
Flow diagram depicting different steps of marker assisted forward breeding to combine multiple biotic-abiotic stress resistance/tolerance in rice Crossing program was initiated in WS2013 which involved crossing of recurrent parent (Swarna+drought, possessing drought tolerant QTLs namely (*qDTY*_*1.1*_, *qDTY*_*3.1*_) with four different donors possessing targeted genes or BB (*Xa4, xa5, xa13, Xa21*), blast (*Pi9*), BPH (*Bph3* and *Bph17*) and GM (*Gm4* and *Gm8*). The number over the chromosome represents the chromosome number of the respective donors. Through several rounds of inter-crossing, IC_3_F_1_ with desirable QTLs/genes combinations were obtained in WS2015. Further, MAFB approach was utilized to combine genes/QTLs for BB, blast, BPH, GM and drought tolerance

The MAFB approach was deployed to transfer 9 genes/QTLs from five different donors in the genetic background of NIL-Swarna+drought (recurrent parent). Crossing program was initiated during wet season (WS) 2013 to combine two QTL for drought (*qDTY*_*1.1*_ and *qDTY*_*3.1*_) already present in the recurrent parent background with one gene for blast (*Pi9*), four genes for BLB (*Xa4*, *xa5*, *xa13*, *Xa21*) and two genes for GM (*Gm4* and *Gm8*) (Fig. 1, Table 1). The recurrent parent was crossed with each of the four donors to develop four F_1_’s cross combinations. Hybridity of F_1_ plants were confirmed using polymorphic markers identified earlier. Positive plants possessing desired combinations were selected for first intercross during dry season (DS) 2014. Two IC_1_F_1_’s obtained (381 seeds from Swarna+drought/IRBL9//Swarna+drought/IRBB60 and 97 seeds from Swarna+drought/Abhaya//Swarna+drought/Aganni) from different inter-cross combinations were confirmed with foreground markers during WS2014. Followed by second round of inter- crossing between Swarna+drought/IRBL9// Swarna+drought/IRBB60 X Swarna+drought/Abhaya//Swarna+drought/Aganni, 1532 IC_2_F_1_ possessing genes for different traits were confirmed using markers linked to different traits during DS2015, among which 127 positive plants were found. Also, Rathu Heenati possessing two genes for BPH (*Bph3* and *Bph17*) was crossed with Swarna+drought in DS2015. In order to bring all 11 genes/QTLs in Swarna+drought background, a third round of intercross between IC_2_F_1_ and F_1_ was attempted and 1284 IC_3_F_1_ were generated. Based on foreground selection 71 plants having all the targeted genes in heterozygous state were advanced to IC_3_F_2_. 10,089 IC_3_F_2_ seeds generated from previous year positive IC_3_F_1_ plants were grown in the field during DS2016. All of these IC_3_F_2_ plants were.

**Table 1:** Genes/QTLs information of the identified pyramided introgression lines

ILs	Genes/QTLs combinations in pyramided lines											Total genes/QTLs
	*Pi9*	*Xa4*	*xa5*	*xa13*	*Xa21*	*Bph3*	*Bph17*	*Gm4*	*Gm8*	*qDTY*_*1.1*_	*qDTY*_*3.1*_	
IL1	+	+	+	–	+	–	+	–	+	+	+	8
IL2	+	+	+	–	–	+	–	+	+	+	+	8
IL3	+	+	+	–	+	+	+	+	–	+	+	9
IL4	–	+	+	–	–	+	+	+	–	+	+	7
IL5	–	+	+	–	+	+	–	+	–	+	+	7
IL6	+	+	+	–	+	+	+	+	+	+	+	10
IL7	–	+	+	–	+	+	–	+	–	+	+	7
Swarna + drought	–	–	–	–	–	–	–	–	–	+	+	2

+ indicate presence of desirable allele of the gene, − indicate absence of desirable allele of the gene

tested for the presence of genes/QTLs, from which 106 IC_3_F_2_ plants possessing seven to ten genes in different combinations were identified, out of which 28 single plants (SPs) were forwarded as families in IC_3_F_3_. About 5763 IC_3_F_3_ seeds were generated during WS2016, selective genotyping of the plants during early stage and then phenotypic selection resulted in selection of 19 positive plants with seven to ten genes. Nineteen families ~ 1100 IC_3_F_4_ plants with seven to ten genes were selectively genotyped followed by phenotypic selection to select SP’s similar or superior to recurrent parents, among which we selected 17 SP’s and forwarded them as families to IC_3_F_5_. During WS2017 for 762 IC_3_F_5_ plants, we followed the same strategy of genotyping and selection of plants from where we selected seven best SP’s with seven to ten genes in different combinations and forwarded seven families to IC_3_F_6_ in DS2018. During WS2018, 552 IC_3_F_7_ plants from seven families were grown at two locations namely, IRRI South Asia Hub Hyderabad and IIRR Rajendranagar for the identification of agronomically superior ILs. During the selection we selected agronomically good performing lines even though few genes were missing. As a result, a total of seven ILs with seven to ten genes/QTLs combinations (*Pi9*+ *Xa4*+ *xa5*+ *Xa21*+ *Bph3*+ *Bph17*+ *Gm4*+ *Gm8+ qDTY*_*1.1*_+ *qDTY*_*3.1*_) were found promising in both locations.

### **Evaluation of Introgression Lines for Agronomic Traits**

Intensive phenotyping was performed from IC_3_F_3_ to IC_3_F_7_ and based on the genotypic data of plants possessing maximum genes, best ILs were forwarded every generation. A total of seven promising ILs were finally characterized in replicated trials in the IC_3_F_7_ generation together with the recurrent parent in two seasons (DS2018 and WS2018). Data from SP’s were recorded for days to 50% flowering (DFF), plant height (PH), panicle length (PL), number of tillers (NT) and single plant yield (SPY) (Table 2). DFF for all the seven ILs in DS2018 and WS2018 were 86 and 97 days respectively, that of the recurrent parent was 121 and 131 days in both seasons. PH of ILs ranged from 67.3 to 85 cm during DS2018 and 64 to 83 cm during WS2018, on the contrary, we found PH of recurrent parent to be 85.3 and 84.3 cm, respectively in DS2018 and WS2018; PL of ILs ranged from 18.3 to 24.3 cm in DS2018 and 19.6 to 24 cm in WS2018 whereas PL of recurrent parent was 21.6 and 22.6 cm in DS2018 and WS2018; NT of ILs ranged from 14 to 22 in DS2018 and 16 to 23 in WS2018 and that of recurrent parent was 19 in both the season. SPY of ILs ranged from 19.4 to 22.4 g during DS2018 and 22.2 to 26.9 during WS2018 which is higher than the recurrent parent (19.6 g in DS2018 and 24.8 g in WS2018) in both the seasons. We also found that these ILs were early maturing (~ 30–35 days) than the recurrent parent with overall good agronomic performance. Since the yield for ILs was higher than the Swarna+drought with early maturity duration, the developed ILs were identified better than the recurrent parent.

**Table 2:** Agronomic performance of introgression lines during DS2018 and WS2018

	DFF^a^ (days)		PH^a^ (cm)		PL^a^ (cm)		NT^a^		SPY^a^ (g)	
Ils	DS2018	WS2018	DS2018	WS2018	DS2018	WS2018	DS2018	WS2018	DS2018	WS2018
IL1	86 ± 0	97 ± 0	80.3 ± 1.2	78 ± 1.1	20.6 ± 0.8	20 ± 1	18 ± 1.2	20 ± 0.8	20.4 ± 1.1	24.1 ± 0.3
IL2	86 ± 0	97 ± 0	67.3 ± 0.6	67 ± 1.1	24.3 ± 0.8	23 ± 1	22 ± 1	23 ± 1.4	20.1 ± 0.6	22.3 ± 1.2
IL3	86 ± 0	97 ± 0	73 ± 1.1	72.3 ± 0.8	19 ± 0.5	19.6 ± 0.6	14 ± 0.8	16 ± 0.8	19.9 ± 2	23.8 ± 0.5
IL4	86 ± 0	97 ± 0	67.3 ± 1.8	69.6 ± 1.2	23 ± 1.5	24 ± 0.5	15 ± 1.4	20 ± 1.2	20.3 ± 0.6	22.2 ± 1
IL5	86 ± 0	97 ± 0	68 ± 1.1	64 ± 1.5	18.3 ± 0.3	20.3 ± 0.6	19 ± 0.5	22. ± 0.6	19.4 ± 0.4	23.5 ± 0.8
IL6	86 ± 0	97 ± 0	82 ± 0.5	83 ± 1	22 ± 1	22.6 ± 1.2	21 ± 0.6	22 ± 0.6	22.4 ± 1.2	26.9 ± 0.5
IL7	86 ± 0	97 ± 0	85 ± 0.5	82.6 ± 1.7	24 ± 0.5	22.6 ± 0.8	22 ± 0.8	23 ± 1.1	22.2 ± 1	25.2 ± 0.3
Swarna + drought	121 ± 1.2	131 ± 0.8	85.3 ± 1.4	84.3 ± 0.3	21.6 ± 1.2	22.6 ± 0.8	19 ± 1	19 ± 0.3	19.6 ± 1.2	24.8 ± 0.6
CD at 95%	0	0	3	3.2	1.5	1.9	9.9	2.6	2.2	1.6

*DFF* days to 50% flowering (days), *PH* plant height (cm), *PL* Panicle length (cm), *NT* Number of tillers, *SPY* Single plant yield (g), *CD* Critical difference

^a^Mean ± SE

### **Phenotypic Evaluation of Introgression Lines for Blast and BLB Resistance**

**Fig. 2:**
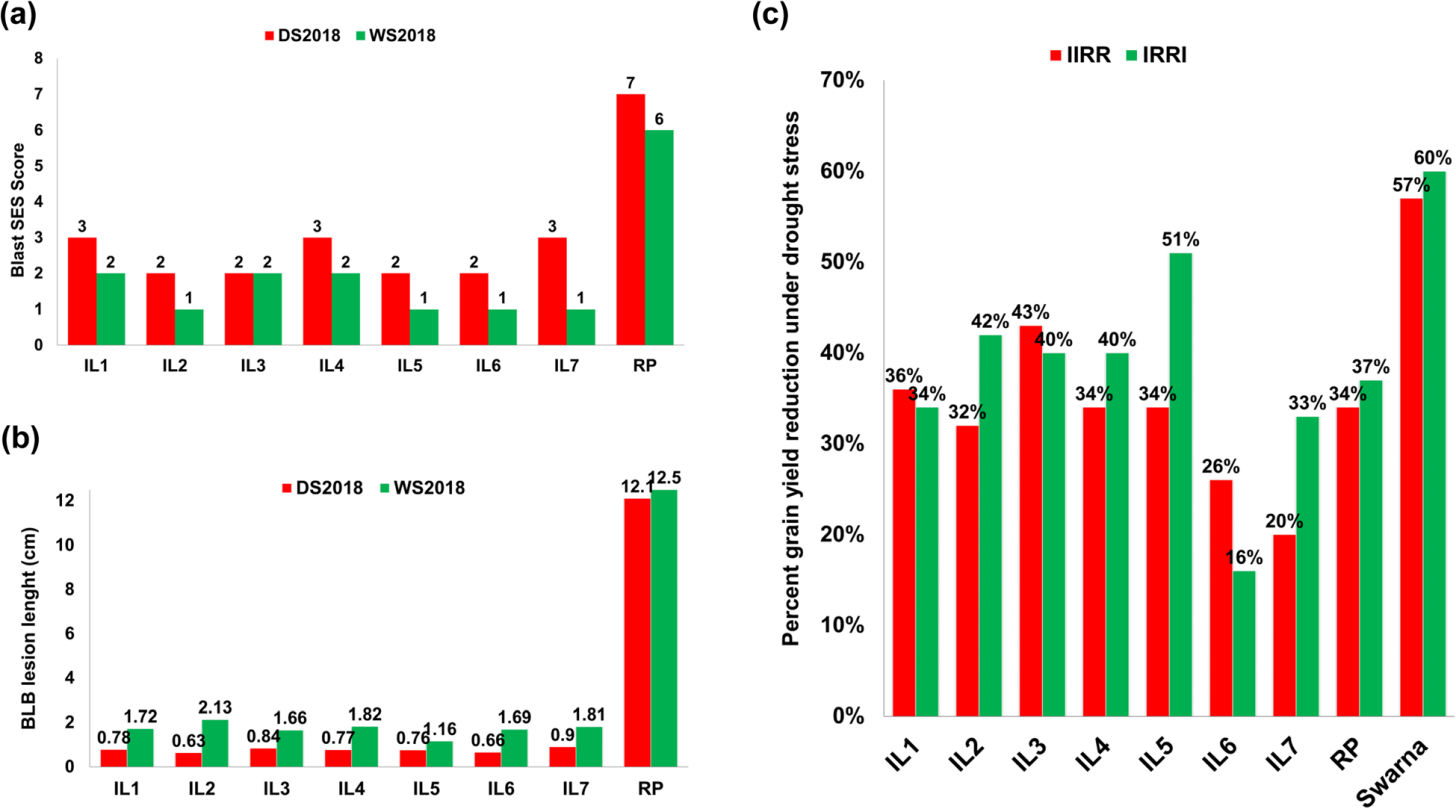
Evaluation of ILs for blast, BLB and drought tolerance Evaluation of promising ILs for the targeted traits during DS2018 and WS2018 (a) performance of ILs and recurrent parent under UBN (uniform blast nursery), (b) performance of ILs for BB resistance by clip inoculation method, (c) performance of ILs for grain yield under reproductive stage drought condition at two locations (IRRI Hyderabad and IIRR Rajendranagar). Performance of ILs was found superior for the targeted traits (blast, BB and drought) in comparison to recurrent parent (RP, Swarna+drought)

During DS2018 and WS2018, all the seven introgressed lines (ILs), recurrent parent, resistant checks (Blast- IRBL9 and BLB- IRBB60) along with susceptible checks (Blast- HR12 and BLB-Naveen) were screened for their reaction to blast and BLB disease. All the ILs expressed resistant (R) to moderately resistant (MR) reaction against blast with SES score 1–3 however the recurrent parent Swarna+drought was found to be highly susceptible with SES score 6–7. For bacterial blight, the average lesion length were recorded for three plants in each ILs and we found that all the ILs expressed higher level of BLB R reaction, showing average lesion length of 1.0–2.0 cm however the recurrent parent Swarna+drought recorded susceptible (S) reaction under field conditions with lesion length of 12 cm (Fig. 2a, b and Table 3).

**Table 3:** Reaction of introgressed lines to blast, BLB and gall midge

ILs	Blast Score		BLB lesion length (cm)		Gall Midge	
	DS2018	WS2018	DS2018	WS2018	Glass house^a^	Hot Spot^b^
IL1	3 (MR)	2 (R)	0.78 (R)	1.72 (R)	MR	MR
IL2	2 (R)	1 (R)	0.63 (R)	2.13 (R)	R	R
IL3	2 (R)	2 (R)	0.84 (R)	1.66 (R)	S	S
IL4	3 (MR)	2 (R)	0.77 (R)	1.82 (R)	S	MR
IL5	2 (R)	1 (R)	0.76 (R)	1.16 (R)	S	S
IL6	2 (R)	1 (R)	0.66 (R)	1.69 (R)	R	R
IL7	3 (MR)	1 (R)	0.9 (R)	1.81 (R)	MR	MR
Swarna + drought	7 (S)	6 (S)	12.1 (S)	12.5 (S)	S	S
IRBL9	2 (R)	2 (R)	–	–	–	–
IRBB60	–	–	0.5 (R)	0.5 (R)	–	–
Abhaya	–	–	–	–	R	R
Aganni	–	–	–	–	R	R

^a^IIRR, Rajendranagar glass house GM screening using Biotype 1

^b^RARS, Warangal hot spot GM screening using Biotype 4 M

Blast (SES, IRRI 2013); 0–2 (Resistant), 3–4 (Moderately Resistant), 5–9 (Susceptible)

BLB lesion length Chen., et al. 2000 R (up to 3 cm), MR (> 3-6 cm), MS (> 6-9 cm), S (> 9 cm)

Gall Midge (SES, IRRI 2002); 0, 1, 3 Resistant and 5, 7, 9 Susceptible

### **Phenotypic Evaluation of Introgression Lines for BPH and GM Resistance**

BPH screening of IC_3_F_6_ ILs possessing *Bph3* and *Bph17* genes was carried out at IIRR, Rajendranagar, during DS2018. Genotyping of the ILs with the targeted markers revealed that IL3, IL4, and IL5 possess both the genes (*Bph3* + *Bph17*); IL2 and IL6 lines possess *Bph3* and IL1 lines possess *Bph17* gene (Tables 1, 3Figure S1). Surprisingly, phenotyping of all the ILs along with the recurrent parents, donor and susceptible check (TN1) for BPH were found to be susceptible. This may have resulted due to emergence of a new biotype during the course of development of these ILs. Gall midge screening of seven ILs was done for biotype I in greenhouse at IIRR, Rajendranagar and biotype 4 M in field following standard protocol at Warangal, Telangana, India, a natural hotspot location. We observed interesting results during this screening process, two ILs (IL2 and IL6) possessing both *Gm4* and *Gm8* genes exhibited R reaction for both biotypes 1 and 4 M. IL1 with *Gm8* and IL7 with *Gm4*, each having only one gene exhibited variable reaction showing MR reaction for both biotypes when present as single gene, IL3 and IL5 showed S reaction along with the recurrent parent and IL4 showed mixed results, i.e. susceptible under glasshouse conditions for biotype1 and MR under hot spot for biotype 4 M, which indicates that the IL4 with *Gm4* may have become susceptible when exposed to more virulent populations such as 4 M (Table 3).

### **Grain Quality Analysis of Introgression Lines**

**Fig. 3:**
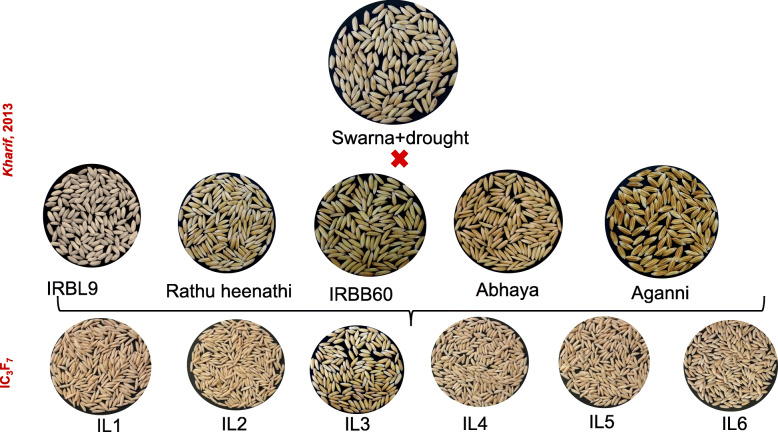
Representative grain picture of the identified ILs with 7–10 genes pyramided in the background of Swarna + drought Grain picture of representative six ILs along with recurrent and all the donor parents used in the breeding program. Grain shape and size of ILs has been affected by the grain type of the donors used in the breeding programs. Grain type of most of the ILs is of medium slender (MS) type except for IL1 and IL2 which are long slender (LS) types

The grain quality data of seven ILs is presented in Fig. 3 and Table 4. Kernel length (KL) of all the seven ILs ranged from 5.5 to 6.9 mm in DS2018 and 5.4 to 7 mm in WS2018 in comparison to 5.7 mm and 5.6 mm of the recurrent parent in DS2018 and WS2018, respectively. Kernel breadth (KB) of ILs ranged from 1.8 to 2.8 mm in DS2018 and 1.8 to 2.9 mm in WS 2018, whereas KB of the recurrent parent was 2.8 and 2.9 mm during DS2018 and WS2018, respectively. Most of the ILs were found to have medium slender (ms) grain type except for IL1 and IL2 which are long slender (ls) grain type. Also, we observed that amylose content (AC) of all the seven ILs were of intermediate to high (AC ranged from 23 to 26% in DS2018 and 23 to 25% in WS2018), close to the recurrent parent (AC 25%). Grain quality analysis for the ILs showed that the grain type varied from recurrent parent because of the five different donors with different grain types used in the crossing program.

**Table 4:** Evaluation of grain quality traits of selected introgression lines during DS2018 and WS2018

ILs	KL (mm)		KB (mm)		LBR		AC (%)	
	DS2018	WS2018	DS2018	WS2018	DS2018	WS2018	DS2018	WS2018
IL1	6.9 ± 0.02 (l)	7 ± 0.18 (l)	2.0 ± 0.06	1.9 ± 0.03	3.5 ± 0.03 (ls)	3.7 ± 0.08 (ls)	25 ± 0.2 (I)	25 ± 0.2 (I)
IL2	6.8 ± 0.11 (l)	6.7 ± 0.09 (l)	2 ± 0.07	1.9 ± 0.03	3.3 ± 0.09 (ls)	3.4 ± 0.10 (ls)	23 ± 0.3 (I)	23 ± 0.2 (I)
IL3	5.5 ± 0.06 (m)	5.6 ± 0.09 (m)	2.7 ± 0.01	2.6 ± 0.03	2.1 ± 0.06 (ms)	2.2 ± 0.06 (ms)	23 ± 0.6 (I)	25 ± 0.3 (I)
IL4	5.9 ± 0.12 (m)	5.8 ± 0.06 (m)	1.8 ± 0.07	1.9 ± 0.03	3.2 ± 0.03 (ms)	3.0 ± 0.06 (ms)	23 ± 0.33 (I)	25 ± 0.3 (I)
IL5	6.0 ± 0.06 (m)	5.9 ± 0.03 (m)	1.9 ± 0.06	1.8 ± 0.06	3.1 ± 0.07 (ms)	3.1 ± 0.13 (ms)	25 ± 0.4 (I)	23 ± 0.6 (I)
IL6	6.1 ± 0.12 (m)	6.0 ± 0.07 (m)	2.0 ± 0.01	2.1 ± 0.03	3.1 ± 0.30 (ms)	2.9 ± 0.06 (ms)	26 ± 0.2 (H)	25 ± 0.3 (I)
IL7	6.0 ± 0.09 (m)	6.1 ± 0.07 (m)	1.9 ± 0.06	2.0 ± 0.06	3.2 ± 0.09 (ms)	3.1 ± 0.06 (ms)	26 ± 0.4 (H)	25 ± 0.1 (I)
Swarna + drought	5.7 ± 0.09 (m)	5.6 ± 0.06 (m)	2.8 ± 0.06	2.9 ± 0.03	2.0 ± 0.09 (mb)	1.9 ± 0.11 (mb)	25 ± 0.2 (I)	25 ± 0.1 (I)
SD	0.42	0.46	0.27	0.24	0.44	0.48	1.29	0.90
CD at 95%	0.25	0.12	0.1	0.08	0.1	0.1	1.7	0.72

*KL* kernel length, KB: kernel breadth, *LB* length-breadth ratio, *AC* amylose content, *I* intermediate amylose content (20–25%), *H* high amylose content (> 25%), SD: standard deviation, *CD* critical difference, *ls* long-slender, *ms* medium slender, *sb* short bold, *mb* medium bold

Grain type classification (combination of KL and LBR) (SES, IRRI 2013)

Kernel length (scale): 1.Extra long (> 7.5 mm), 3.long (6.6–7.5 mm), 5.medium (5.51–6.6 mm), 7.short (5.5 mm or < 5.5 mm)

Length-breadth ratio (scale): 1. Slender (> 3), 3. medium (2.1–3), 5. bold (1.1–2), 7. round (< 1.1)

### **Phenotypic Evaluation of Introgression Lines for Drought Tolerance**

**Fig. 4:**
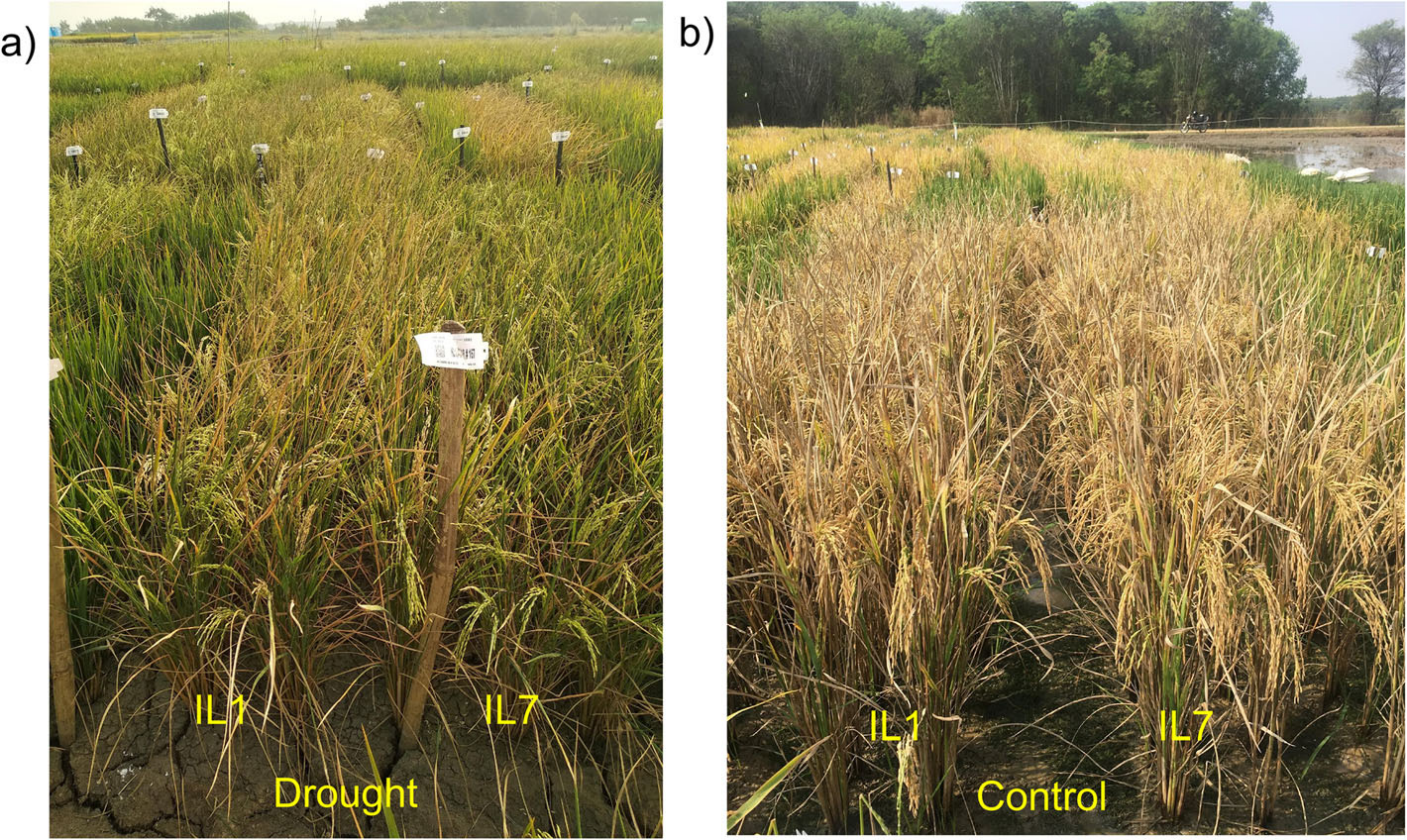
Field performance of high yielding selected ILs Performance of IL1 (8 QTLs/genes combination) and IL7 (8 QTLs/genes combination) having different combinations have been compared in drought and control conditions during WS2018

Recurrent parent used in the crossing program possess drought QTLs in its background, but in order not to miss these important drought QTLs during multiple genes/QTLs introgression in recurrent parent background, we confirmed all the plants after each crosses for the presence of drought QTLs - *qDTY*_*1.1*_ and *qDTY*_*3.1*_. The seven selected ILs were screened for drought tolerance (reproductive stage) at two locations of IRRI Hyderabad and IIRR Rajendranagar during WS2018 (Table 5). Yield reductions of ILs were observed between 20% to 43% at IRRI, Hyderabad and between 16% to 50% at IIRR, Rajendranagar compared with non-stress control yield at both the locations respectively. The performance of ILs was found similar to the recurrent parent Swarna+drought (~ 34.1% yield reduction at IRRI, Hyderabad and 37% yield reduction at IIRR, Rajendranagar) (Fig. 2c, Fig. 4) but significantly higher over Swarna with yield reduction of 60% and 57.3% at IRRI and IIRR, respectively.

**Table 5:** Screening of introgression lines under reproductive stage drought stress (WS2018)

ILs	IIRR (kg/ha)		IRRI (kg/ha)	
	Control^a^	Drought^a^	Control^a^	Drought^a^
IL1	3948 ± 62.1	2546 ± 40.2	4497 ± 70.9	2968 ± 46.9
IL2	4579 ± 53.2	3093 ± 35.8	5815.5 ± 67.5	3398 ± 39.4
IL3	6246 ± 125.6	3546 ± 71.2	5318 ± 106.9	3180 ± 63.8
IL4	4765 ± 72.8	3160 ± 48.3	4627.5 ± 70.9	2796 ± 42.7
IL5	3190 ± 66.9	2100 ± 44.3	4472.5 ± 93.8	2212 ± 46.4
IL6	2393 ± 50.6	1761 ± 37.4	4226 ± 89.4	3558 ± 75.3
IL7	3561 ± 42.6	2834 ± 34	5532 ± 66.4	3692 ± 44.4
Swarna + drought	5501 ± 55.8	3627 ± 26.6	3517 ± 73.7	2216 ± 62.2
Swarna	5395 ± 71.3	2305 ± 58.1	4596 ± 49.4	1816 ± 56.8
CD at 95%	268.7	171.0	305.2	195.9

*CD* critical difference

^a^Mean ± SE

### **Principal Component Analysis (PCA) of Introgression Lines**

**Fig. 5:**
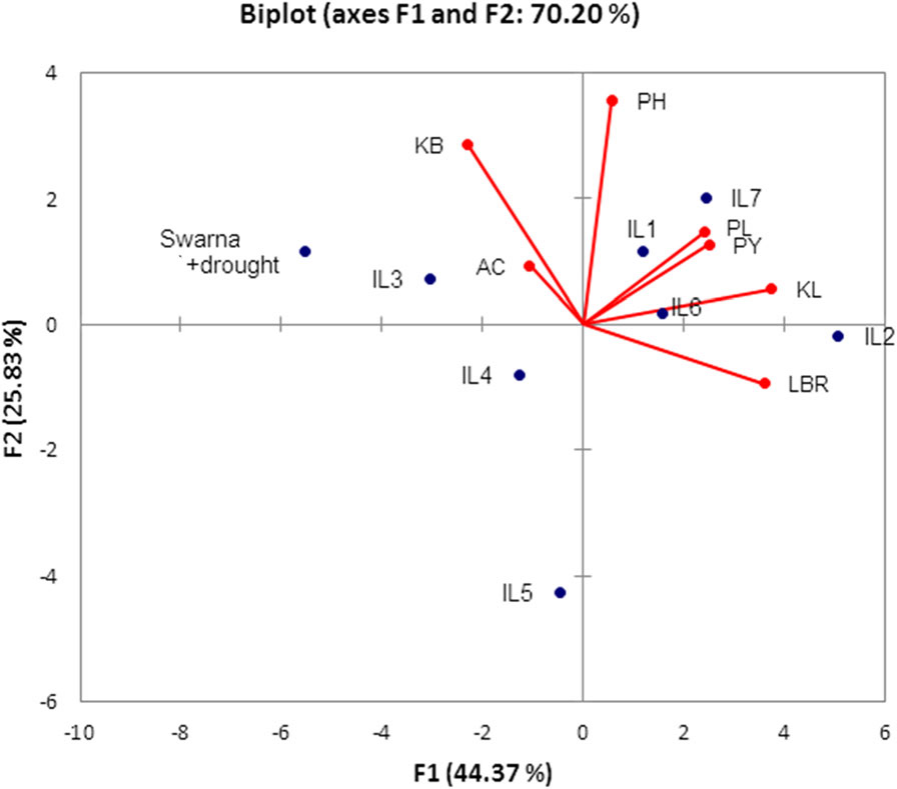
Principal component analysis of ILs PCA of ILs for different grain quality traits (KL, KB, LBR, AC) and agronomic traits (PH, PL, PY) during WS2018 showed that IL1, IL6 and IL7 with different genes/QTLs combination perform better than recurrent parent

The performance of seven ILs has been predicted using PCA analysis (Fig. 5). PCA analysis revealed that IL1 with *Pi9*+ *Xa4*+ *xa5*+ *Xa21*+ *Bph17*+ *Gm8*+ *qDTY*_*1.1*_+ *qDTY*_*3.*1_ (eight genes), IL6 with *Pi9*+ *Xa4*+ *xa5*+ *Xa21*+ *Bph3*+ *Bph17*+ *Gm4*+ *Gm8+ qDTY*_*1.1*_+ *qDTY*_*3.*1_ (ten genes) and IL7 with *Pi9*+ *Xa4*+ *xa5*+ *Bph3+ Gm4*+ *qDTY*_*1.1*_+ *qDTY*_*3.*1_ (seven genes) showed yield advantage under stress over recurrent parent. It is very clear from the figure that PH, PL, KL, and PY are in the same direction and are positively related to each other for these ILs. We further conclude from PCA analysis that three ILs (IL1, IL6 and IL7) were superior to recurrent parent.

## **Discussion**

Most of the high yielding rice varieties are susceptible to biotic and abiotic stresses. Conventional breeding has been successfully employed to develop several varieties with higher yield but developing multiple stress tolerant varieties using this approach is very cumbersome. Recent identification of resistance/tolerance source and trait associated marker has opened the opportunity to introgress many traits together with the help of MAS. MAS has played a very important role in improvement of rice varieties with two or more genes/QTLs (Singh et al. 2001; Kottapalli et al. 2010; Pradhan et al. 2015) in a shorter time. Looking to the importance of MAS, we have used time and cost effective MAFB to stack multiple genes/QTLs for BLB, BL, GM, BPH and drought in recurrent parent Swarna+drought.

This breeding strategy utilized both molecular and conventional approaches to incorporate multiple traits followed by stringent selection during each generation. Large population sizes during early generation were maintained for selecting plants with good phenotypic performance together with different gene combinations. In the present study genes for BLB, blast, GM, BPH and QTLs for drought traits have been successfully introgressed in different combinations. We also observed that the grain quality traits of recurrent parents except grain type was successfully retained. Some ILs were found to show better grain quality traits because of stringent phenotypic selection during early generations as well as use of elite donors for many of the traits.

Several *R* genes for blast are known, among them *Pi9* gene confer broad-spectrum resistance against diverse isolates of blast pathogen in India and because of rare compatibility of blast isolates to *Pi9* gene and its broad-spectrum nature we have introgressed only one gene for blast. ILs were checked with markers and tested for blast reaction under field condition in uniform blast nursery (UBN). The segregating materials showed a wide range of reaction among which only resistant/moderately resistant lines were transplanted in the main field and the rest of the susceptible materials were discarded. We also faced severe practical problems with the *Pi9* marker amplification and observed that the results were obtained only from the freshly isolated DNA in addition to freshly diluted markers. Four ILs homozygous for *Pi9* gene exhibited a resistant reaction with SES score ranging from 1 to 3. Previously, Khanna et al. (2015) had successfully demonstrated introgression of seven blast genes in Pusa Basmati 1 (PB1) and found that among seven genes NILs carrying *Pi9* were most effective against virulent races of blast diseases especially in hot stop locations. Interestingly, we found that in three of our ILs, though missing the resistant alleles of *Pi9* gene but showed resistant reaction under UBN. This could be due to the utilization of other donors such as Abhaya and IRBB60 used in the current breeding program. It was also reported that Abhaya was found to be resistant against blast (blast SES score ranged from 1 to 3 with different isolates) (Sinha et al. 2017) isolates. Similarly, IRBB60 was also reported to have moderately resistant reaction with blast (with blast SES score of 3) (Chukwu et al. 2020).

While working with BLB, the challenge appears to be more due to the presence of the number of genetically distinct virulent *Xoo* strains in different geographical locations (Midha et al. 2017). In order to achieve broad spectrum of resistance against BLB, the most practical and feasible method used by scientists is to combine two or more genes rather than single genes to overcome the issue of pathogens turning virulent (Mundt et al. 1999). Among the various available BLB genes, *Xa21* gene is reported to provide broad-spectrum resistance against *Xoo* races. However for durable resistance, we used four different BLB genes (*Xa4*, *xa5*, *xa13,* and *Xa21*) in the introgression program, because this combination was found superior to all other gene combinations (Dokku et al. 2013a, Dokku et al. 2013b, Das and Rao 2015). In contrast, Swarna, the original parent of the improved Swarna+drought was highly susceptible to BLB. ILs pyramided with these *R* genes showed a highly resistant (HR) reaction from season to season using *Xoo* isolate DX-020 with lesion length < 3 cm. ILs with BLB genes have higher resistance scores and reduced lesion length (.63 to 2.13 cm) than recurrent parent (12.1 to 12.5 cm). High level of resistance response was reported with the introgression of these genes in previous studies (Singh et al. 2001; Joseph et al. 2004; Sundaram et al. 2008; Rajpurohit et al. 2011; Pradhan et al. 2015).

For BPH, several resistance genes have been identified, however major concern is the emergence of new biotypes/mixed populations of different biotypes because of immigration of new biotypes from neighboring countries (Choi et al. 1979; Yeo et al. 1998). We have introgressed *Bph3* and *Bph17* in our study, however on screening these ILs we did not find our lines to show higher levels of resistance. There is a dire need to first identify the population structure over years and then evaluate the identified genes (Jena et al. 2017) at hotspot locations and suggest the gene/combinations of genes to be introgressed for stable resistant reaction against BPH.

GM gene introgression in several popular rice varieties such as Swarna, Samba Mahsuri have been quite successful (Kumar et al. 2000; Biradar et al. 2004; Kumaravadivel et al. 2006; Himabindu et al. 2010). Losses resulting from gall midge infestation have resulted in huge crop loss during past years. In previous studies, pyramiding of more than one gene worked well for getting durable resistance. In current study, two gall midge gene *Gm4* and *Gm8* were introgressed. ILs with *Gm8* genes alone or in combination worked well and provided good resistance. It was also observed significant variation in the disease response of the ILs with similar gene combinations. For instance, IL3, IL4, IL5 and IL7 possess similar gene combinations for GM (*Gm4*) however, IL3, IL4 and IL5 are found to be susceptible in comparison to moderate resistance of IL7. This could be due to the difference in the genome recovery and background interaction of genes/QTLs of the ILs which resulted in different phenotypic responses. The effect of epistatic interaction, genetic background on the differential phenotypic response of introgression lines is evidenced in few studies (Basavaraj et al. 2010; Singh et al. 2012; Yadav et al. 2019).

Ensuring food security in the context of climate change is the foremost challenge and improvement of rice varieties that can resist adverse conditions such as drought and submergence is of utmost priority. With a wide range of changes happening in climate, ensuring stable production and productivity of rice for millions of poor farmers of Asia is a major concern as millions of hectares of the monsoon-based rice crop is severely affected by numerous biotic and abiotic stresses year after year. Several drought QTLs have been identified (Vikram et al. 2011; Venuprasad et al. 2009; Ghimire et al. 2012; Yadaw et al. 2013; Mishra et al. 2013) in the past years. We made consistent efforts to maintain drought QTLs (*qDTY*_*1.1*_ and *qDTY*_*3.1*_) in the recurrent parent in order to retain its tolerance to drought even after introgression of multiple genes/ QTLs.

The pyramiding of genes was accomplished using a systematic forward breeding approach and at the end of the first cycle, plants having different gene/QTL but similar/better to the recurrent parent in morphology and grain shape were selected. Together with phenotypic selection, practice of foreground selection has resulted in identification of lines closer to the parent in morphology with different combinations of genes. In the present study, using the same recurrent parent in multiple crosses and inter-crossing program with intensive genotypic-phenotypic selection and grain quality-based selection has helped in regaining plants of recurrent parent type to a great extent despite using five different donors and not following backcross breeding strategy. Furthermore, in the present study the pyramided lines were evaluated for their closeness according to their agro-morphological and grain quality traits with the recurrent parent Swarna+drought.

Three ILs of Swarna+drought (IL1, IL6, and IL7) possessing seven to ten genes hold huge potential in rice breeding as these ILs show high levels of resistance/tolerance against multiple stresses especially blast, BLB, GM and drought. After the release of these forward breeding products as varieties, the problem of occurrence of both biotic and abiotic stresses can be addressed simultaneously.

The rising number of pathogenic variants of different diseases and the appearance of new and active insect biotypes necessitates immediate attention. Using conventional breeding, introgression of huge numbers of different genes/QTLs would be next to impossible, but with the recent advances in markers technology has led this to a successful mission with significant savings in time, space, labor, and money. This new generation rice lines developed using a forward breeding approach with seven to nine different genes/QTLs in mega rice variety can prove to be very useful in the molecular breeding program. This major breakthrough with cost-effective investment is a clear-cut demonstration of the efforts of plant breeders to combat multiple biotic and abiotic stresses in one go without any adverse effect on the agronomic and grain quality traits which will help in increasing both production and productivity of rice in the years to come.

## **Conclusion**

Development of broad-spectrum resistance against diseases like blast, BLB and insects like GM, BPH in the Indian subcontinent is a foremost challenge due to the multiplicity of the agro-climatic zones where rice is cultivated under the presence of a number of genetically distinct virulent strains/biotypes in different geographical areas of India. In addition, under the climate change scenario, tolerances to drought assume great significance to rice. The current study has demonstrated that the deployment of appropriate gene or gene combinations against each biotic and abiotic stress can help develop new ILs with durable and broad-spectrum resistance/tolerance. However, it may be noted that cases like BPH need to be evaluated carefully to find out the appropriate population and relevant gene combinations for different regions. The developed ILs will be of great use in the future rice breeding programs and breeders can use them suiting to their needs as varieties or as donors for introgressing multiple genes/QTLs.

## **Material and Methods**

### **Planting Material and Breeding Scheme**

Swarna+drought, rice NIL developed at the International Rice Research Institute (IRRI), Philippines was used as the recurrent parent for the pyramiding of multiple QTL/genes associated with different abiotic and biotic stresses. Swarna+drought is a popular *indica* cultivar in different parts of the country because of its high yielding, medium slender (ms) grains and its capacity to mitigate drought stress.

Although Swarna is a popular variety in many regions it is susceptible to several biotic stresses. To address this problem MAFB approach (Fig. 1) was initiated in WS2013 at International Rice Research Institute-South Asia Hub (IRRI-SAH), Hyderabad (78^°^ 16^′^ longitude, 17^°^ 32^′^ latitude and 540 m above sea level). Looking towards the severity caused by different stresses we planned to introgress different biotic genes for blast, BLB, BPH and GM together with drought QTLs already present in recurrent parent. The DNA markers linked to these traits have been used based on published literature (Table S1). The donors used in this study are IRBB60 for BLB resistance genes *Xa4*, *xa5*, *xa13,* and *Xa21,* IRBL9 for blast resistance gene *Pi9*, Rathu Heenati a Srilankan landrace for two BPH resistance genes namely *Bph3* and *Bph17*, for gall midge resistance two cultivars namely Abhaya and Aganni for *Gm4* and *Gm8* genes*,* respectively were used (Table S2). Initially, single cross F_1_’s were developed by making simple cross combinations, which was started during WS2013 as Swarna+drought/IRBB60, Swarna+drought/IRBL9, Swarna+drought/Abhaya and Swarna + drought/Aganni.

The MAFB breeding scheme was initiated in WS2013 with the making of simple crosses among.

all the donors. F_1_’s resulting from two rounds of intercrosses in different gene combinations were brought together in one genetic background during WS2015. During this process of introgression all IC_3_F_1_’s in each season were confirmed using tightly linked polymorphic SSR markers for the targeted QTLs/genes. After genotyping from IC_3_F_2_ to IC_3_F_7_ generation followed by intensive phenotypic selection SP’s with phenotypes close to recurrent or superior to recurrent parents were selected and forwarded until WS2018.

### **Genotyping**

DNA was isolated by a TPS method adapted from (IRRI-Japan collaborative research project) Dr. Yohei Koide and modified by Lenie A. Quiatchon (Kim et al. 2016). Leaf samples were collected for all the donors and recipients 2 weeks after transplantation. The primers used in this introgression program were selected based on the available published reports as discussed earlier. The annealing temperature for all markers was standardized and polymerase chain reaction (PCR) component (Genie) was used for PCR analysis using freshly isolated DNA (Table S3). The separated PCR products were visualized under UV light using Syngene imager (Syngene, USA).

### **Parental Polymorphism Survey**

For the parental polymorphism survey, parents involved in this study with Swarna+drought as recurrent parent and donors are IR96321–1447-561-B-1 (*qDTY*_*1.1*_, *qDTY*_*3.1*_- drought), IRBL9 (*Pi9*- blast), IRBB60 (*Xa4*, *xa5*, *xa13*, and *Xa21*- BLB), Rathu Heenati (*Bph3* and *Bph17*- BPH), Abhaya- (*Gm4*- GM) and Aganni (*Gm8*- GM). Several SSR/STS markers were used to select the polymorphic markers associated with target QTLs/genes between recipient and donor parents for MAS.

### **Agronomic Trait Evaluation**

Extensive phenotypic selection for the advancement of introgressed segregating material during every generation was carried out after the genotypic confirmation of plants at initial stage. Plants with maximum positive genes were tagged in the field and their phenotypic performance was evaluated. The selected seven ILs were grown in 6 rows of 3 m length along with checks and recurrent parents. The row to row distance was 0.2 m and hill to hill distance was 0.15 m. Standard agronomic packages were performed. Phenotyping involved data collection for DFF, PH, NT, NP, grain type, plant type, test weight (TW), yield per plant. Data of five plants from each ILs were recorded, future which average was computed and the average data were used to represent PH, NT, NP, yield per plant. In addition to this, photographs were taken for superior ILs during every season showing good agronomic performance with maximum number of genes together with yield comparable to our recurrent parent. Descriptive statistics was performed using SPSS software for the phenotypic traits (PH, PL, NT, TW, GY) recorded for ILs from DS2018 and WS2018. Based on the information collected for these ILs during the conduct of experiment it became easy to understand the performance of different ILs.

### **Evaluation of Grain Quality Traits**

Superior ILs with good agronomic traits were also analyzed for grain quality traits at IRRI Hyderabad during DS2018 and WS2018, using the standard evaluation method for grain quality rice. Completely dried grains with relative humidity (RH) 12–13% were taken for grain quality trait estimation. We recorded traits like KL, KB, length/ breadth (LBR) ratio (SES, IRRI 2013) and (AC) of five milled grains from each IL further which average was computed. Vernier caliper was used to measure KL, KB and LB ratio from milled complete grains. For AC estimation cut grains method was utilized (Agasimani et al. 2013).

### **Screening for Blast Resistance**

A local isolate of *Magnaporthe oryzae* (SPI-40) from IIRR, Rajendranagar (Madhan Mohan 2011), were used to screen the donor and recurrent parents from IC_3_F_6_ and IC_3_F_7_ ILs during DS2018 and WS2018 for blast resistance under in vivo conditions following uniform blast nursery (UBN) method at IRRI Hyderabad. All the ILs seedlings were planted in 2 rows of 50 cm each. The pathogen strains were revived and multiplied on oatmeal agar plate for inoculum production. The young seedlings at four-leaf stage were inoculated with the fungal conidial suspension at a concentration of 1 × 10^5^ conidia/ml, and high RH > 80% was maintained for disease development. Inoculated seedlings were monitored for the development of blast lesions 1 week after inoculation. The plants were scored and evaluated on a 0–9 scale as per IRRI-SES scale (SES, IRRI 2013).

### **Screening for BLB Resistance**

A virulent isolate of the bacterial blight pathogen, *Xanthomonas oryzae* pv. *Oryzae* (*Xoo*) from Indian Institute of Rice Research (IIRR) *Xanthomonas* collection DX-020 were used to screen the donor and recurrent parents along with ILs of Swarna+drought for BB resistance under field conditions in IC_3_F_6_ and IC_3_F_7_ generation DS2018 and WS2018*.* The *Xoo* strains were grown on peptone sucrose agar media for inoculum production. Bacterial growth was scraped from all the plates and re-suspended in sterilized distilled water. The rice plants were inoculated at maximum tillering stage (40–45 days old) with freshly prepared inoculum following clip inoculation method with sterilized scissors (Kauffman 1973). Approximately 5–7 uppermost leaves were inoculated in each plant, and disease reaction was scored after 14 days after inoculation. Three single plants from each ILs in IC_3_F_6_ and IC_3_F_7_ were screened under field conditions following the same procedure. The lesion lengths were measured on all inoculated leaves 14 days post-inoculation when lesions were stable. The average lesion length of three longest lesions from individual plants was calculated. A plant was classified as R if the average lesion length was less than 0–3 cm, MR if the lesion was > 3–6 cm, moderately susceptible (MS) if the lesion was > 6–9 cm, and S if the lesion was longer than > 9 cm (Chen et al. 2000).

### **Screening for Brown Planthopper Resistance/ Tolerance**

BPH biotype 4 from IIRR Rajendranagar was used to screen BPH under controlled glasshouse conditions with TN1 (Kalode et al. 1975) susceptible rice variety. Newly hatched nymphs or adults were utilized for screening IC_3_F_7_ ILs during DS2018. IC_3_F_7_ ILs along with S check parents TN1 and PTB33 as R checks were evaluated following standard protocol (Kalode et al. 1975). Seedlings were sown in a tray in three replications with two border rows of the S check (TN1) and one row of R check PTB33 in center, 10 days after sowing seedlings in 3–4 leaf stage were infested with 6–8 instars nymphs per seedling. Standard Evaluation System (SES) method was used for evaluation of damage and score on a scale of 0–9 (SES, IRRI 2002).

### **Screening for Gall Midge Resistance/ Tolerance**

Gall midge screening was done for two biotypes. Screening for Biotype 1 was done under controlled conditions in the glasshouse at IIRR, Rajendranagar and Biotype-4 was done at natural hotspot location Warangal. All the IC_3_F_7_ ILs were screened for Gall midge biotype 1 (GMB1) under controlled conditions during WS2018. Ten days old seedlings along with checks TN1 (S-check), Abhaya and Aganni (R-check) were planted in plastic pots for GM screening as per standard protocol (Lakshmi et al. 2006). The classification of lines into R and S classes was recorded when the susceptible plants showed 90–100% plant damage with the emergence of galls. The Entries were classified as resistant/susceptible based on plant damage; plants with 0–10% plant damage were R, and those greater than 10% were S. For hotspot screening, all the IC_3_F_7_ ILs along with checks were raised under field conditions at Warangal, Telangana. All the agronomic practices were followed except the application of any insecticide. Disease symptoms were recorded 30–50 days after planting based on presence of silver soot damage. 0–9 SES scale used for scoring the plants under field as well controlled condition (SES, IRRI 2002).

### **Screening for Drought Tolerance**

IC_3_F_7_ ILs was screened for a reproductive stage severe drought during WS2018 in open field condition. Drought under reproductive-stage stress in low land areas is critical which severely affects yield. We selected drought tolerant and susceptible checks together with our ILs for screening under reproductive stage drought and non-stress condition in Augmented RCBD design. The selected seven ILs along with recurrent parent and susceptible checks were grown in the field during WS2018. Each entry was grown in 4 rows with 3 m length. Hill to hill distance was 0.15 m and row to row was 0.2 m. 1.10 m length of piezometer or water table tube was installed by keeping 1.0 m in below the soil surface and 10 cm above the ground in the drought screening field for measuring the water table depth throughout the crop cycle. Irrigation was stopped 1 month after transplanting. Life saving irrigation was given when susceptible checks started to show severe leaf rolling symptoms in the morning at 10 am. Several cycles of stress imposition and application of life saving irrigation were repeated until harvesting. When all the susceptible checks started to show severe leaf rolling symptoms with minimum probability to recover upon watering, the fields were flood-irrigated and completely drained out after 24 h of irrigation. At physiological maturity the phenotypic observations and post harvest data were recorded for the ILs, parents and checks.

## Supplementary information

**Additional File 1: Table S1.** Gene based/linked markers used for foreground selection for blast, BLB, BPH, GM and drought resistance/tolerance genes/QTLs and their validation in the developed lines **Table S2.** List of parents, markers used for parental polymorphism and foreground selection in introgression lines of Swarna+drought **Table S3.** Allele size of different gene based/linked markers linked to different trait of interest.

**Additional File 2: Figure S1.** Screening of the ILs for BLB, blast and gall midge. (a) screening for bacterial leaf blight resistance (b) screening for blast resistance (c) Screening for BPH reaction of ILs under glass house condition (d) BPH screening of ILs under hotspot location.

## Data Availability

The relevant supplementary data has been provided with the manuscript. The developed NILs can be obtained from IRRI with proper material-transfer agreement (MTA) and/or a standard material-transfer agreement (SMTA).

## References

[CR1] Agasimani S, Selvakumar G, Joel AJ, Ganesh Ram S (2013). A simple and rapid single kernel screening method to estimate amylose content in rice grains. Phytochem Anal.

[CR2] Akram R, Fahad S, Masood N, Rasool A, Ijaz M, Ihsan MZ, Maqbool MM, Ahmad S, Hussain S, Ahmed M, Kaleem S (2019). Plant growth and morphological changes in Rice under abiotic stress. Advances in Rice research for abiotic stress tolerance.

[CR3] Amante-Bordeos A, Sitch LA, Nelson R, Dalmacio RD, Oliva NP, Aswidinnoor H, Leung H (1992). Transfer of bacterial blight and blast resistance from the tetraploid wild rice *Oryza minuta* to cultivated rice, *Oryza sativa*. Theoret Appl Genet.

[CR4] Arunakumari K, Durgarani CV, Satturu V, Sarikonda KR, Chittoor PDR, Vutukuri B, Laha GS, Nelli APK, Gattu S, Jamal M, Prasadbabu A (2016). Marker-assisted pyramiding of genes conferring resistance against bacterial blight and blast diseases into Indian rice variety MTU1010. Rice Sci.

[CR5] Ashkani S, Rafii MY, Shabanimofrad M, Foroughi M, Azizia P, Akhtar MS, Sahebi M, Harun AR, Nasehi A (2015). Multiplex SSR–PCR approaches for semi-automated genotyping and characterization of loci linked to blast disease resistance genes in rice. C R Biol.

[CR6] Babujee L, Gnanamanickham SS (2000). Molecular tools for characterization of rice blast pathogen (*Magnaporthe grisea*) population and molecular marker-assisted breeding for disease resistance. Curr Sci.

[CR7] Basavaraj SH, Singh VK, Singh A, Singh A, Singh A, Anand D, Yadav S, Ellur RK, Singh D, Krishnan SG, Nagarajan M (2010). Marker-assisted improvement of bacterial blight resistance in parental lines of Pusa RH10, a superfine grain aromatic rice hybrid. Mol Breed.

[CR8] Bellard C, Bertelsmeier C, Leadley P, Thuiller W, Courchamp F (2012). Impacts of climate change on the future of biodiversity. Ecol Lett.

[CR9] Bentur JS, Pasalu IC, Sarma NP, Prasada Rao U, Mishra B (2003) Gall midge resistance in rice. Directorate of Rice Research, Hyderabad, vol 20

[CR10] Biradar SK, Sundaram RM, Thirumurugan T, Bentur JS, Amudhan S, Shenoy VV, Mishra B, Bennett J, Sarma NP (2004). Identification of flanking SSR markers for a major rice gall midge resistance gene Gm1 and their validation. Theoret Appl Genet.

[CR11] Busungu C, Taura S, Sakagami JI, Ichitani K (2016). Identification and linkage analysis of a new rice bacterial blight resistance gene from XM14, a mutant line from IR24. Breed Sci.

[CR12] Chen S, Lin XH, Xu CG, Zhang Q (2000). Improvement of bacterial blight resistance of ‘Minghui 63’, an elite restorer line of hybrid rice, by molecular marker-assisted selection. Crop Sci.

[CR13] Choi SY, Heu MM, Lee JO (1979). Varietal resistance to the brown planthopper in Korea. International Rice research institute. Brown planthopper: threat to rice production in Asia, Los Baños.

[CR14] Chukwu SC, Rafii MY, Ramlee SI, Ismail SI, Oladosu Y, Kolapo K, Musa I, Halidu J, Muhammad II, Ahmed M (2020). Marker-assisted introgression of multiple resistance genes confers broad spectrum resistance against bacterial leaf blight and blast diseases in Putra-1 rice variety. Agron.

[CR15] Das A, Soubam D, Singh PK, Thakur S, Singh NK, Sharma TR (2012). A novel blast resistance gene, *Pi54rh* cloned from wild species of rice, Oryza rhizomatis confers broad spectrum resistance to *Magnaporthe oryzae*. Funct Integr Genomic.

[CR16] Das G, Rao GJ (2015). Molecular marker assisted gene stacking for biotic and abiotic stress resistance genes in an elite rice cultivar. Front Plant Sci.

[CR17] Das G, Rao GJ, Varier M, Prakash A, Prasad D (2018). Improved Tapaswini having four BB resistance genes pyramided with six genes/QTLs, resistance/tolerance to biotic and abiotic stresses in rice. Sci Rep.

[CR18] Dilla-Ermita CJ, Tandayu E, Juanillas VM, Detras J, Lozada DN, Dwiyanti MS, Cruz CV, Mbanjo EG, Ardales E, Diaz MG, Mendioro M (2017). Genome-wide association analysis tracks bacterial leaf blight resistance loci in rice diverse germplasm. Rice.

[CR19] Dokku P, Das KM, Rao GJN (2013). Genetic enhancement of host plant-resistance of the Lalat cultivar of rice against bacterial blight employing marker-assisted selection. Biotechnol Lett.

[CR20] Dokku P, Das KM, Rao GJN (2013). Pyramiding of four resistance genes of bacterial blight in Tapaswini, an elite rice cultivar, through marker-assisted selection. Euphytica.

[CR21] Ghimire KH, Quiatchon LA, Vikram P, Swamy BM, Dixit S, Ahmed H, Hernandez JE, Borromeo TH, Kumar A (2012). Identification and mapping of a QTL (*qDTY*_*1.1*_) with a consistent effect on grain yield under drought. Field Crop Res.

[CR22] Hasan MM, Rafii MY, Ismail MR, Mahmood M, Rahim HA, Alam MA, Ashkani S, Malek MA, Latif MA (2015). Marker-assisted backcrossing: a useful method for rice improvement. Biotechnol Biotechnol Equip.

[CR23] He C, Xiao Y, Yu J, Li J, Meng Q, Qing X, Xiao G (2019). Pyramiding *Xa21*, *Bph14*, and *Bph15* genes into the elite restorer line Yuehui9113 increases resistance to bacterial blight and brown planthopper in rice. J Crop Prot.

[CR24] Himabindu K, Suneetha K, Sama VSAK, Bentur JS (2010). A new rice gall midge resistance gene in the breeding line CR57-MR1523, mapping with flanking markers and development of NILs. Euphytica.

[CR25] Jena KK, Hechanova SL, Verdeprado H, Prahalada GD, Kim SR (2017). Development of 25 near-isogenic lines (NILs) with ten BPH resistance genes in rice (*O*. *sativa L*.): production, resistance spectrum, and molecular analysis. Theoret Appl Genet.

[CR26] Jeung JU, Heu SG, Shin MS, Vera Cruz CM, Jena KK (2006). Dynamics of *Xanthomonas oryzae* pv. *oryzae* populations in Korea and their relationship to known bacterial blight resistance genes. Phytopathol.

[CR27] Joseph M, Gopalakrishnan S, Sharma RK, Singh VP, Singh AK, Singh NK, Mohapatra T (2004). Combining bacterial blight resistance and basmati quality characteristics by phenotypic and molecular marker-assisted selection in rice. Mol Breed.

[CR28] Kalode MB, Viswanathan PR, Seshu DV (1975). Standard test to characterise host plant resistance to Brown Plant hopper in Rice. Indian J Plant Prot.

[CR29] Kauffman HE (1973). An improved technique for evaluating resistance of rice varieties to *Xanthomonas oryzae*. Plant Dis Rep.

[CR30] Khanna A, Sharma V, Ellur RK, Shikari AB, Krishnan SG, Singh UD, Prakash G, Sharma TR, Rathour R, Variar M, Prashanthi SK (2015). Development and evaluation of near-isogenic lines for major blast resistance gene (s) in basmati rice. Theoret Appl Genet.

[CR31] Khush GS (2005). What it will take to feed 5.0 billion rice consumers in 2030. Plant Mol Biol.

[CR32] Khush GS, Jena KK (2009). Current status and future prospects for research on blast resistance in rice (*Oryza sativa* L.). Adv Genet Genomics Control Rice Blast Dis.

[CR33] Kim SR, Yang J, An G, Jena KK (2016). A simple DNA preparation method for high quality polymerase chain reaction in rice. Plant Breed Biotech.

[CR34] Kottapalli KR, Narasu ML, Jena KK (2010). Effective strategy for pyramiding three bacterial blight resistance genes into fine grain rice cultivar, samba Mahsuri, using sequence tagged site markers. Biotechnol Lett.

[CR35] Kumar A, Bhandarkar S, Pophlay DJ, Shrivastava MN (2000). A new gene for gall midge resistance in rice accession Jhitpiti.

[CR36] Kumar A, Sandhu N, Dixit S, Yadav S, Swamy BPM, Shamsudin NAA (2018). Marker-assisted selection strategy to pyramid two or more QTLs for quantitative trait-grain yield under drought. Rice.

[CR37] Kumar VA, Balachiranjeevi CH, Naik SB, Rekha G, Rambabu R, Harika G, Pranathi K, Hajira SK, Anila M, Kousik M, Kale R (2017). Marker-assisted pyramiding of bacterial blight and gall midge resistance genes into RPHR-1005, the restorer line of the popular rice hybrid DRRH-3. Mol Breed.

[CR38] Kumaravadivel N, Uma MD, Saravanan PA, Suresh H (2006) Molecular marker-assisted selection and pyramiding genes for gall midge resistance in rice suitable for Tamil Nadu region In abstracts-2nd IRC:257

[CR39] Lakshmi PV, Amudhan S, Bindu KH, Cheralu C, Bentur JS (2006). A new biotype of the Asian rice gall midge Orseolia oryzae (Diptera: Cecidomyiidae) characterized from the Warangal population in Andhra Pradesh, India. Int J Trop Insect Sc.

[CR40] Madhan Mohan K (2011) Molecular characterization of pathogenic variability of Pyricularia grisea (Rice Blast fungus) (Doctoral dissertation, Ph. D. thesis Faculty of Biotechnology, Jawaharlal Nehru Technological University, Hyderabad)

[CR41] Midha S, Bansal K, Kumar S, Girija AM, Mishra D, Brahma K, Laha GS, Sundaram RM, Sonti RV, Patil PB (2017). Population genomic insights into variation and evolution of X*anthomonas oryzae* pv. *oryzae*. Sci Rep.

[CR42] Mishra KK, Vikram P, Yadaw RB, Swamy BM, Dixit S, Cruz MTS, Maturan P, Marker S, Kumar A (2013). *qDTY*_*12.1*_: a locus with a consistent effect on grain yield under drought in rice. BMC Genet.

[CR43] Mundt CC, Ahmed HU, Finckh MR, Nieva LP, Alfonso RF (1999). Primary disease gradients of bacterial blight of rice. Phytopathol.

[CR44] Muthu V, Abbai R, Nallathambi J, Rahman H, Ramasamy S, Kambale R, Thulasinathan T, Ayyenar B, Muthurajan R (2020). Pyramiding QTLs controlling tolerance against drought, salinity, and submergence in rice through marker assisted breeding. PLoS One.

[CR45] Pang Y, Chen K, Wang X, Wang W, Xu J, Ali J, Li Z (2017). Simultaneous improvement and genetic dissection of salt tolerance of rice (*O*. *sativa L*.) by designed QTL pyramiding. Front Plant Sci.

[CR46] Pradhan SK, Nayak DK, Mohanty S, Behera L, Barik SR, Pandit E, Lenka S, Anandan A (2015). Pyramiding of three bacterial blight resistance genes for broad-spectrum resistance in Deepwater rice variety, Jalmagna. Rice.

[CR47] Qing D, Dai G, Zhou W, Huang S, Liang H, Gao L, Gao J, Huang J, Zhou M, Chen R, Chen W (2019). Development of molecular marker and introgression of *Bph3* into elite rice cultivars by marker-assisted selection. Breed Sci.

[CR48] Qu S, Liu G, Zhou B, Bellizzi M, Zeng L, Dai L, Han B, Wang GL (2006). The broad-spectrum blast resistance gene *Pi9* encodes a nucleotide-binding site–leucine-rich repeat protein and is a member of a multigene family in rice. Genetics.

[CR49] Rajpurohit D, Kumar R, Kumar M, Paul P, Awasthi A, Basha PO, Puri A, Jhang T, Singh K, Dhaliwal HS (2011). Pyramiding of two bacterial blight resistance and a semidwarfing gene in type 3 basmati using marker-assisted selection. Euphytica.

[CR50] Sandhu N, Dixit S, Swamy BP, Raman A, Kumar S, Singh SP, Yadaw RB, Singh ON, Reddy JN, Anandan A, Yadav S (2019). Marker assisted breeding to develop multiple stress tolerant varieties for flood and drought prone areas. Rice.

[CR51] SES, IRRI (2002). Standard evaluation system.

[CR52] SES, IRRI (2013). Standard evaluation system.

[CR53] Singh S, Sidhu JS, Huang N, Vikal Y, Li Z, Brar DS, Dhaliwal HS, Khush GS (2001). Pyramiding three bacterial blight resistance genes (*xa5*, *xa13* and *Xa21*) using marker-assisted selection into indica rice cultivar PR106. Theoret Appl Genet.

[CR54] Singh VK, Singh A, Singh SP, Ellur RK, Choudhary V, Sarkel S, Singh D, Krishnan SG, Nagarajan M, Vinod KK, Singh UD (2012). Incorporation of blast resistance into “PRR78”, an elite basmati rice restorer line, through marker-assisted backcross breeding. Field Crop Res.

[CR55] Sinha SK, Sarawgi AK, Singh AK (2017). Genetic analysis of blast resistant gene in rice (*Oryza sativa* L.) cultivars. ORYZA-An Int J Rice.

[CR56] Sitch LA, Amante AD, Dalmacio RD, Leung H (1989). *Oryza minuta*, a source of blast and bacterial blight resistance for rice improvement. In 2. International symposium on genetic manipulation in crops, El Batan (Mexico), IRRI, 29-31 Aug 1988.

[CR57] Suh JP, Cho YC, Won YJ, Ahn EK, Baek MK, Kim MK, Kim BK, Jena KK (2015). Development of resistant gene-pyramided Japonica rice for multiple biotic stresses using molecular marker-assisted selection. Plant Breed Biotech.

[CR58] Sundaram RM, Vishnupriya MR, Biradar SK, Laha GS, Reddy GA, Rani NS, Sarma NP, Sonti RV (2008). Marker assisted introgression of bacterial blight resistance in samba Mahsuri, an elite indica rice variety. Euphytica.

[CR59] Swathi G, Rani CVD, Md J, Madhav MS, Vanisree S, Anuradha C, Kumar NR, Kumar NAP, Kumari KA, Bhogadhi SC, Ramprasad E (2019). Marker-assisted introgression of the major bacterial blight resistance genes, *Xa21* and *xa13*, and blast resistance gene, *Pi54*, into the popular rice variety, JGL1798. Mol Breed.

[CR60] Venuprasad R, Dalid CO, Del Valle M, Zhao D, Espiritu M, Cruz MS, Amante M, Kumar A, Atlin GN (2009). Identification and characterization of large-effect quantitative trait loci for grain yield under lowland drought stress in rice using bulk-segregant analysis. Theoret Appl Genet.

[CR61] Vikram P, Swamy BM, Dixit S, Ahmed HU, Cruz MTS, Singh AK, Kumar A (2011). *qDTY*_*1.1*_, a major QTL for rice grain yield under reproductive-stage drought stress with a consistent effect in multiple elite genetic backgrounds. BMC Genet.

[CR62] Wang H, Gao Y, Mao F, Xiong L, Mou T (2019). Directional upgrading of brown planthopper resistance in an elite rice cultivar by precise introgression of two resistance genes using genomics-based breeding. Plant Sci.

[CR63] Wang Y, Jiang W, Liu H, Zeng Y, Du B, Zhu L, He G, Chen R (2017). Marker assisted pyramiding of *Bph6* and *Bph9* into elite restorer line 93–11 and development of functional marker for *Bph9*. Rice.

[CR64] Wu Y, Xiao N, Yu L, Pan C, Li Y, Zhang X, Liu G, Dai Z, Pan X, Li A (2015). Combination patterns of major R genes determine the level of resistance to the *M*. *oryzae* in rice (*Oryza sativa* L.). PLoS One.

[CR65] Yadav S, Sandhu N, Majumder RR, Dixit S, Kumar S, Singh SP, Mandal NP, Das SP, Yadaw RB, Singh VK, Sinha P (2019). Epistatic interactions of major effect drought QTLs with genetic background loci determine grain yield of rice under drought stress. Sci Rep.

[CR66] Yadaw RB, Dixit S, Raman A, Mishra KK, Vikram P, Swamy BM, Cruz MTS, Maturan PT, Pandey M, Kumar A (2013). A QTL for high grain yield under lowland drought in the background of popular rice variety Sabitri from Nepal. Field Crop Res.

[CR67] Yasmin S, Hafeez FY, Mirza MS, Rasul M, Arshad HM, Zubair M, Iqbal M (2017). Biocontrol of bacterial leaf blight of rice and profiling of secondary metabolites produced by rhizospheric *Pseudomonas aeruginosa* BRp3. Front Microbiol.

[CR68] Yeo US, Jin YD, Kim HY, Park NB, Lim SJ, Hwang HG, Kim SC, Shon JK (1998). Linkage between shikimic dehydrogenase isomerase and a brown planthopper (*Nilaparvata lugens*) resistance gene in a japonica rice Milyang 4. Korean J Breed.

[CR69] Yugander A, Sundaram RM, Ladhalakshmi D, Hajira SK, Prakasam V, Prasad MS, Madhav MS, Babu VR, Laha GS (2017). Virulence profiling of *Xanthomonas oryzae* pv. *oryzae* isolates, causing bacterial blight of rice in India. Eur. J. Plant Pathol.

[CR70] Zheng W, Wang Y, Wang L, Ma Z, Zhao J, Wang P, Zhang L, Liu Z, Lu X (2016). Genetic mapping and molecular marker development for *Pi65* (t), a novel broad-spectrum resistance gene to rice blast using next-generation sequencing. Theoret Appl Genet.

