# Supplementary material for: Marker Assisted Forward Breeding to Combine Multiple Biotic-Abiotic Stress Resistance/Tolerance in Rice
Source: Rice (N Y). 2020 May 29;13:29. doi: 10.1186/s12284-020-00391-7 (PMC7260318; doi:10.1186/s12284-020-00391-7)
Supplement: Supplementary file 1 — Additional File 1: Table S1. Gene based/linked markers used for foreground selection for blast, BLB, BPH, GM and drought resistance/tolerance genes/QTLs and their validation in the developed lines Table S2. List of parents, markers used for parental polymorphism and foreground selection in introgression lines of Swarna+drought Table S3. Allele size of different gene based/linked markers linked to different trait of interest. [file 12284_2020_391_MOESM1_ESM.docx]

**Table S1**: Gene based/linked markers used for foreground selection for BL, BLB,, GM and drought resistance/tolerance genes/QTLs and their validation in the developed lines

| **Target traits** | **Linked gene** | **Markers** | **Chr** | **Primer sequence** | **Annealing temperature (°C )** | **Extension (min)** | **Reference** |
| --- | --- | --- | --- | --- | --- | --- | --- |
| Blast | *Pi9* | *Pi9STS2* | 6 | TGCTGACTGCTTGCTATTCGT | 53/45 | 60 | Qu *et al*., 2006 |
|  |  |  |  | GTACTTCATCTTGAGCGACGCAA |  |  |  |
| BLB | *Xa4* | *Xa4* | 11 | ATCGATCGATCTTCACGAGG | 54/35 | 30 | Ma *et al*.,1999 |
|  |  |  |  | TGCTATAAAAGGCATTCGG |  |  |  |
|  | *xa5* | *xa5DRR* | 5 | TAGCTGCTGCCGTGCTGTGC | 55/45 | 30 | Zhang *et al*.,1996; Singh *et al*.,2011; Dokku *et al*., 2013b |
|  |  |  |  | AATATTTCAGTGTGCATCTC |  |  |  |
|  | *xa13* | *xa13prom* | 8 | GGCCATGGCTCAGTGTTTAT | 58/35 | 40 | Yoshimura *et al*.,1995; Dokku *et al*., 2013b |
|  |  |  |  | GAGCTCCAGCTCTCCAAATG |  |  |  |
|  | *Xa21* | *pTA248* | 11 | AGACGCGGAAGGGTGGTTCCCGGA | 57/45 | 60 | Ronald *et al*.,1992; Dokku *et al*., 2013b |
|  |  |  |  | AGACCGGTAATCGAAAGATGAAA |  |  |  |
| BPH | *Bph3* | RM586 | 4 | ACCTCGCGTTATTAGGTACCC | 56/35 | 45 | [Jairin *et al*., 2007a](https://www.ncbi.nlm.nih.gov/pmc/articles/PMC5895560/#CR13) |
|  |  |  |  | GAGATACGCCAACGAGATAC |  |  |  |
|  | *Bph17* | RM8213 | 6 | AGCCCAGTGATACAAAGATG | 55/35 | 30 | [Sun *et al*., 2005](https://www.ncbi.nlm.nih.gov/pmc/articles/PMC4908088/#CR83) |
|  |  |  |  | GCGAGGAGATACCAAGAAAG |  |  |  |
| GM | *Gm4* | *Gm4LRR* | 8 | TAGGTTGGCAGACCTTTTCG | 57/30 | 60 | Nair *et al*.,1996 |
|  |  |  |  | GTCAAGATCATCCTCGTAGCG |  |  |  |
|  | *Gm8* | *Gm8PRP* | 8 | TCATGTTGTGCAGATCAACC | 52/45 | 30 | Kumar *et al*., 2001 |
|  |  |  |  | AGCCATATGAAAACCACCAA |  |  |  |
| Drought | *qDTY_1.1_* | RM431 | 1 | TCCTGCGAACTGAAGAGTTG | 56/45 | 30 | Dixit et al., 2014 |
|  |  |  |  | GAGCAAAACCCTGGTTCAC |  |  |  |
|  | *qDTY_3.1_* | RM168 | 3 | TGCTGCTTGCCTGCTTCCTTT | 58/45 | 30 | Vikram *et al*., 2011, Ghimire *et al*., 2012 |
|  |  |  |  | GAAACGAATCAATCCACGGC |  |  |  |

BLB: bacterial leaf blight, BPH: brown plant hopper, GM: gall midge, Chr: chromosome

**Table S2:** List of parents, markers used for parental polymorphism and foreground selection in introgression lines of Swarna+drought

| **Trait** | **Donors** | **QTLs/Genes/** | **No. of markers used** | **Markers Used** | **Markers for foreground selection** |
| --- | --- | --- | --- | --- | --- |
| **BL** | IRBL9 | *Pi9* | 8 | *Pi91F, Pi92F, Pi93F, Pi9*, RM527, RM3330, RM224, RM12705 | *Pi9STS2* |
| **BLB** | IRBB4 | *Xa4* | 17 | *Xa4*, RM27317, RM27318, RM27328, RM27333, RM27336, RM27340, RM27354, RM27356, RM27322, RM5538, RM7228, RM114, RM224, RM277, RM201, RM7654 | *Xa4 gene* |
|  | IRBB60 | *xa5* | 6 | *xa5*, xa5DRR, RM122, RM159, RM13, RM159 | *xa5DRR* |
|  |  | *xa13* | 2 | *xa13 , xa13pau* | *xa13 gene* |
|  |  | *Xa21* | 1 | *Xa21* (*pTA248*) | *pTA248* |
| **GM** | Abhaya | *Gm4* | 5 | RM22550, RM219, RM547, RM444, RM22565, *Gm4LRR* | *Gm4LRR* |
|  | Aganni | *Gm8* | 5 | RM22683, RM22685, RM22687, RM22710, | *Gm8PRP* |
|  |  |  |  | RM22711*, Gm8PRP* |  |
| **BPH** | Rathu Heenathi | *Bph3* | 9 | RM589, RM586, RM588, RM190, RM261, RM401, RM190, RM469, RM204 | RM589(LFM), RM586(PM), RM190(RFM) |
|  |  | *Bph17* | 3 | RM8213, RM5953, RM16556 | RM8213(LFM), RM16556(RFM ) |

Where, LFM: left flanking marker, RFM: right flanking marker

**Table S3:** Allele size of different genes/QTLs based and linked markers

| **Target traits** | **Linked gene** | **Markers** | \| **Recurrent allele (bp)** \|  \| \| --- \| --- \| | \|  \| **Donor allele (bp)** \| \| --- \| --- \| |
| --- | --- | --- | --- | --- | --- | --- | --- | --- |
| **BL** | *Pi9* | *Pi9STS2* | 210 | 1100 |
| **BLB** | *Xa4* | *Xa4 gene* | 200 | 190 |
|  | *xa5* | *xa5DRR* | 300 | 200 |
|  | *xa13* | *xa13prom* | 270 | 350 |
|  | *Xa21* | *pTA248* | 220 | 990 |
| **BPH** | *Bph3* | RM586 | 160 | 300 |
|  | *Bph17* | RM8213 | 180 | 200 |
| **GM** | *Gm4* | *GM4LRR* | 550 | 210 |
|  | *Gm8* | *GM8PRP* | 350 | 1100 |
| **Drought** | *qDTY_1.1_* | RM431 | 280 | 380 |
|  | *qDTY_3.1_* | RM168 | 220 | 140 |
