# Supplementary material for: Marker Assisted Forward Breeding to Combine Multiple Biotic-Abiotic Stress Resistance/Tolerance in Rice
Source: Rice (N Y). 2020 May 29;13:29. doi: 10.1186/s12284-020-00391-7 (PMC7260318; doi:10.1186/s12284-020-00391-7)
Supplement: Supplementary file 2 — Additional File 2: Figure S1. Screening of the ILs for BLB, blast and gall midge. (a) screening for bacterial leaf blight resistance (b) screening for blast resistance (c) Screening for BPH reaction of ILs under glass house condition (d) BPH screening of ILs under hotspot location. [file 12284_2020_391_MOESM2_ESM.pptx]

## Slide 1
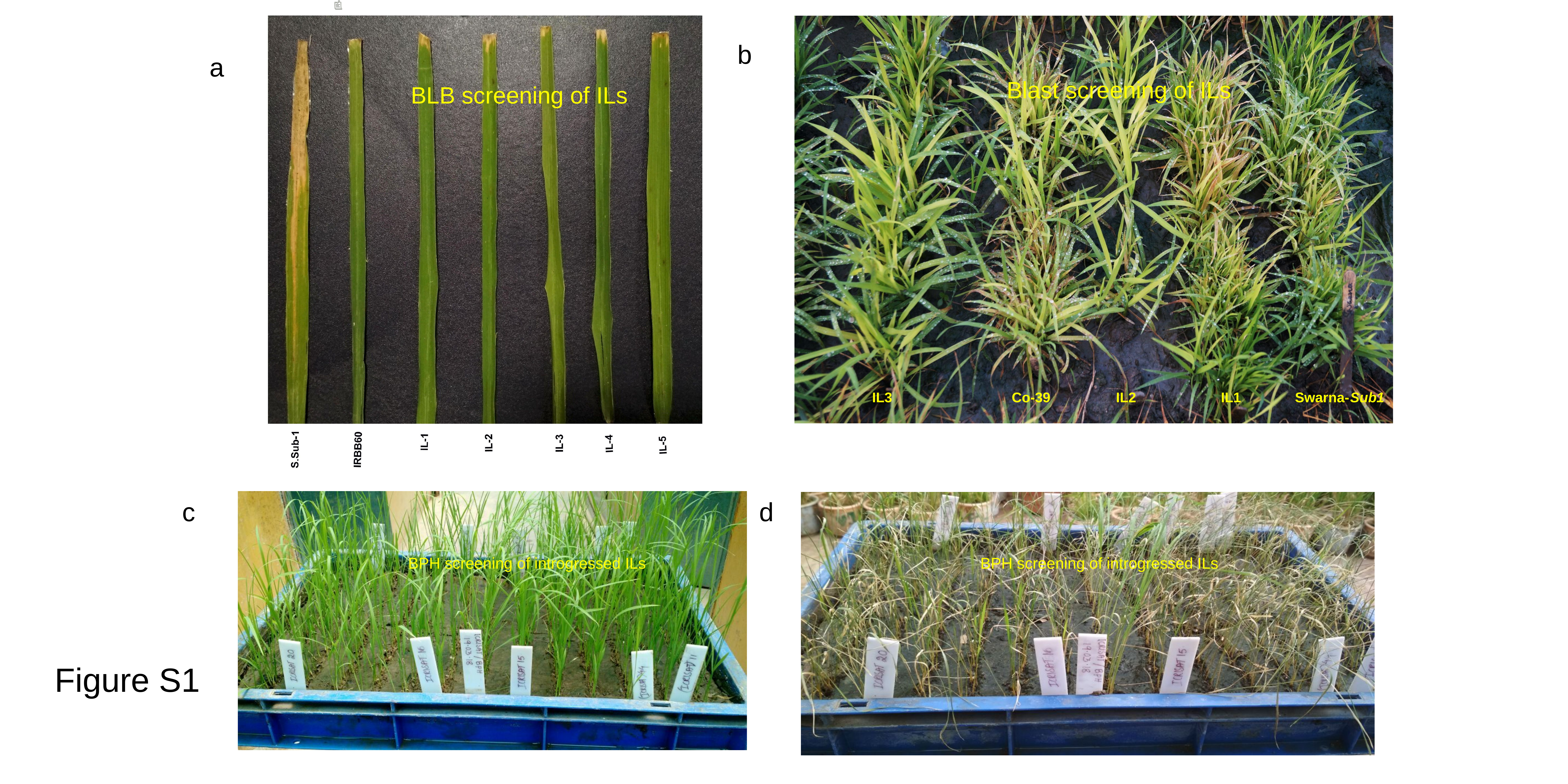

IL3 Co-39 IL2 IL1 Swarna-Sub1
b
a
Blast screening of ILs
BLB screening of ILs
c
d
BPH screening of introgressed ILs
BPH screening of introgressed ILs
Figure S1
